# A Systematic Review and Meta-Analysis of the Prevalence of Hallux Valgus in the General Population

**DOI:** 10.7759/cureus.42739

**Published:** 2023-07-31

**Authors:** Khalid M Alkhalifah, Mohammad M Almotiri, Abdulmajeed E Alharbi, Alanoud Alrashidi, Ibrahim K Aldhali, Osama S Alsaqry, Khalid H Alharbi, Ismail Almogbil

**Affiliations:** 1 Unaizah College of Medicine and Medical Sciences, Qassim University, Buraydah, SAU; 2 Department of Surgery, Unaizah College of Medicine and Medical Sciences, Qassim University, Buraydah, SAU

**Keywords:** forefoot, prevalence, bunion, hv, hallux valgus

## Abstract

The objective of this systematic review was to determine the prevalence of hallux valgus (HV) in the general population by conducting a review and meta-analysis of existing studies. Published articles on the incidence of HV were systematically searched and evaluated on reputable medical databases such as PubMed. The keywords “the prevalence of hallux valgus and/or bunions”were used to create the search syntax on the various databases. Data were gathered on prevalence, population under study, and methodology.

A total of 11 articles that met the search criteria were identified and included in this review for a total of 10,886 participants across the studies. The pooled prevalence of HV across the studies was3.75 (95% confidence interval = 0.388-0.517). Therefore, the prevalence varied widely across the studies reviewed. Prevalence was observed to be high among females and increased with risk factors such as body mass index, which were identified as significant across the studies. This review was limited by insufficient data and the lack of a standard HV diagnosis method. Therefore, a standard HV diagnosis tool is recommended.

## Introduction and background

Hallux valgus (HV) is a ubiquitous chronic orthopedic condition that affects the forefoot. HV occurs when the first metatarsophalangeal joint (hallux) deviates laterally toward the other joints, and thus the medial prominence of the first metatarsal head [1]. This deviation is associated with functional limitations, pain, and reduced quality of life for affected individuals [2,3] Understanding the predominance of HV is crucial for effective management and resource allocation.

Exploring the epidemiology of HV unveils a complex interplay of genetic, anatomical, and environmental factors. Despite the increased attention to this condition and the rich historic literature, discrepancies in the actual prevalence of HV in the general population persist [4]. Numerous medical reports have only been able to estimate the prevalence of HV. Reports indicate a significantly higher prevalence among females and the elderly population. In the United States and the United Kingdom, reports indicate a prevalence of 1% and 28.4%, respectively [5]. The prevalence of HV in Saudi Arabia was 5.47% [3]. However, results from numerous reports vary widely, leaving a knowledge gap in the literature to be bridged. By conducting a systematic review and meta-analysis, this review aims to synthesize the existing evidence, offering a comprehensive assessment of HV prevalence and enhancing our understanding of this prevalent foot pathology.

The ability to estimate the impact of HV is constrained by the lack of robust clinical data on the condition [5]. However, recent advancements in diagnostic techniques, including radiographic measurements, clinical assessments, and validated questionnaires, have facilitated the accurate detection and classification of HV cases [6,7]. Our study aims to comprehensively appraise the prevalence of HV by consolidating the available literature. We account for methodological variations, consider different gender and age groups, and ultimately facilitate evidence-based decision-making.

## Review

Methodology

Data Sources

We searched various electronic databases (PubMed, MEDLINE, CINAHL, and Scopus) for publications on HV dated 2019 or older. Keywords and a wide range of Medical Subject Headings such as “hallux valgus” or “bunion” were combined with key epidemiological terms such as “survey” and “systematic review.” The Boolean word “OR” was also used. The title and abstract search were carefully screened and those that met the selection criteria were downloaded. Please refer to the Appendices for the full search syntax and the truncations used.

The full texts were reviewed followed by a check of citations. Additional scholarly resources were obtained from Google Scholar. The reference lists of all articles obtained were scrutinized to identify government-sponsored research articles or theses, obsolete articles (i.e., articles older than 1985), and articles that lacked abstracts.

Study Selection Criteria

**Figure 1:**
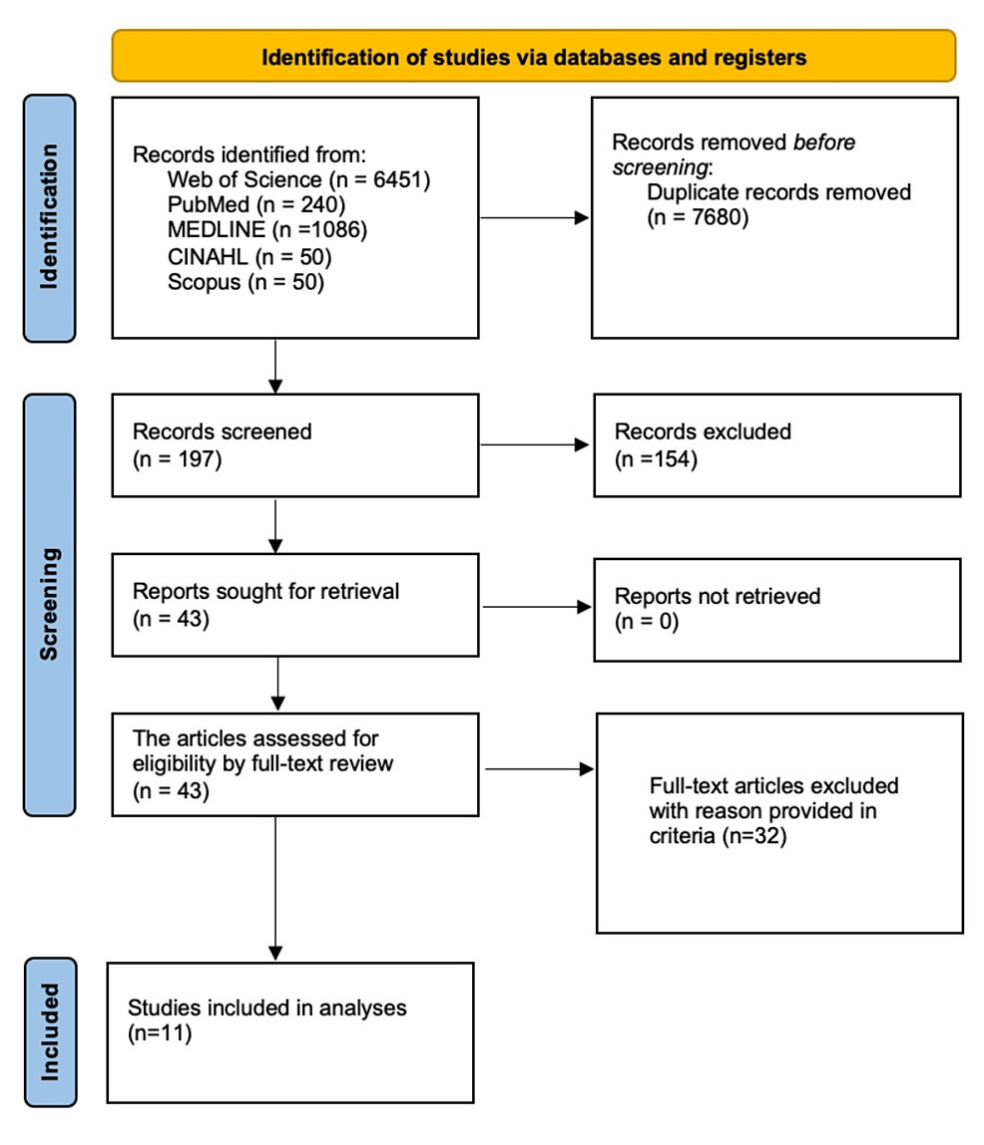
Preferred Reporting Items for Systematic Review and Meta-Analysis (PRISMA) flowchart.

The eligibility criteria for this study were studies that involved the prevalence of HV and/or bunions. We sourced full-text articles where possible and performed a comprehensive eligibility criterion based on HV diagnoses, both in clinical settings or self-reported HV cases, a pre-determined study design, and medical reports based on original quantitative data on the prevalence of bunions. Studies that combined HV prevalence with other conditions, intervention studies, and medical surveys involving specific groups of patients or certain disease cohorts (such as diabetes) were excluded. Studies were chosen regardless of the study designs adopted. Priority was accorded to articles written in the English language. The screening process involved two researchers. The Preferred Reporting Items for Systematic Reviews and Meta-Analyses (PRISMA) guidelines were followed (Figure 1).

Quality Assessment

Epidemiological Appraisal Instrument (EAI) was used by an independent third party to check and score this review for quality. The EAI is a tool designed to assess the quality and methodological rigor of epidemiological studies, including systematic reviews. It provides a structured framework for evaluating the various aspects of a study’s design, conduct, and reporting. In this quality assessment, items that were not relevant to the study designs described above were omitted, yielding a 17-item count. All titles, authors, and other details were removed to identify all articles before rating. Scoring was as follows: “Yes” (score = 2), “No” (score = 0), NA (removed from assessment). Quality assessment was agreed on before scoring by all parties to resolve any disagreements. Each study was then categorized as either “High” or “Low” in the quality assessment based on individual scores.

Data Extraction and Abstraction

Data were obtained using a data collection form by the first author. Regular meetings were held to resolve any disagreements and address any queries. Important research characteristics as defined in the keywords sections of this search criteria were extracted alongside prevalence data for each population under study. The author’s first name, the year of publication, and other demographic characteristics of respondents such as average age and prevalence percentage were acquired.

Results

Database Search

The initial database search produced a total of 7,877 records, of which 7,680 records were eliminated based on the title and abstract screening text. The remaining 197 records were assessed, and 154 records were removed as they involved children, were published in a language other than English, and lacked relevance to the topic. A total of 43 records fulfilled the study’s search criteria and were assessed for eligibility through a full-text review. The assessment excluded a further 32 studies, leaving a total of 11 studies for inclusion in this review.

Study Characteristics

The characteristics of each study were significantly different across the selected articles for this review. As shown in Table 1, the variation was based on geographical location, sample size, age, and prevalence of HV in the study population. Overall, 25% of the studies under review were conducted in the United States, and 16% in Japan, while the remaining studies were done in Saudi Arabia, the United Kingdom, Nigeria, Spain, and France. Most of the studies were performed in a clinical environment while the rest involved self-administered questionnaires to the target population. Three of the 11 studies were published from 2020 to the time of this publication. Two studies were published in 2007, while the most recent one was published in 2021. There was a wide variation in sample sizes, with the largest being 4,249 and the smallest being 66 individuals.

**Table 1:** Characteristics of the included studies. CI: confidence interval

Author	Country	Sample (n)	Age group	Prevalence	Lower 95% CI	Upper 95% CI
Alkhaibary et al., 2019 [3]	Saudi Arabia	420	15 and above	43%	0.384	0.512
Nishimura et al., 2014 [8]	Japan	403	Above 65 years	30%	0.383	0.511
Thomas et al., 2020 [9]	USA	66	11–64 years	30%	0.321	0.444
Piqué-Vidal et al., 2007 [10]	USA	1,174	5 and above	56%	0.402	0.532
González-Martín et al., 2017 [11]	Spain	1,837	>15 years	39%	0.408	0.537
Roddy et al., 2008 [12]	UK	4,249	>30 years	28%	0.415	0.545
Senga et al., 2021 [13]	Japan	604	>50 years	27%	0.391	0.52
Owoeye et al., 2011 [14]	Nigeria	970	10–25 years	15%	0.399	0.528
Cho et al., 2009 [15]	Korea	563	40–69 years	65%	0.39	0.519
Nguyen et al., 2010 [16]	USA	600	20–64 years	42%	0.391	0.52

The pooled prevalence of HV across studies was 3.75 (95% confidence interval (CI) = 0.388-0.517). The pooled variance of HV prevalence across the studies was 1.735223 at 95% CI. The pooled variance was relatively large and statistically significant, indicating substantial heterogeneity across the 11 studies. The prevalence of HV, therefore, varied significantly across the various samples in the studies.

Quality Assessment

The quality assessment procedure conducted during this review revealed that most studies used a simple description of the study population’s demographic traits such as age, gender, and body mass index (BMI). In most of the studies, data on HV prevalence was obtained through questionnaires (self-administered or otherwise), and 25% of the studies utilized a random sampling technique. Overall, 90% of the studies alluded to a higher HV prevalence among females, with all other risk factors held constant.

Meta-Analysis

A meta-analysis was performed to establish aggregated predominance rates using the random-effects model, which provides an average estimation across the studies weighted by sample size. The prevalence estimates were only pooled from studies with similar characteristics due to the variability of research populations. We divided age into the following three major groups for this subgroup analysis of age: children (under 18 years), adults (18-65 years), and the elderly (above 65 years). Summary statistics were obtained in the form of a prevalence proportion, i.e., the number of participants with a positive HV confirmation as a percentage of the entire study sample. This method allowed for the calculation of the standard error.

Forest Plot

**Figure 2:**
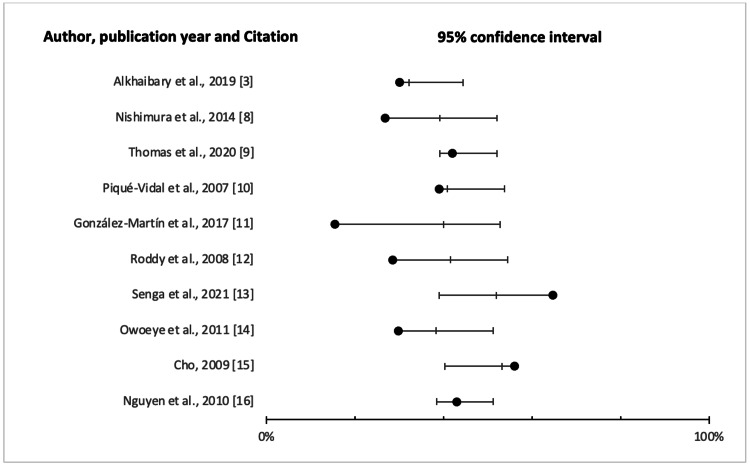
Forest plot depicting the odds ratio.

Figure 2 illustrates the overall prevalence across the studies. Each study is indicated by the point estimate of the prevalence and the 95% CI by random effects.

Discussion

Despite the apparent high prevalence of HV among the study populations in this review, HV is widely ignored and dismissed as a mere anomaly [7]. This review presented a high variation in the prevalence rates of HV owing to several factors such as demographic characteristics of the study population (age, gender, BMI) [8] and the mode of HV diagnosis employed in the study (self-examination/reporting versus clinical observation of HV). As a result, the prevalence of HV was lower in studies whose method of examination was self-administered questionnaires. Studies that used self-reporting methods included a significantly higher sample size [9-12]. Therefore, this review alludes to the fact that considerable under-reporting of HV may be a significant factor in studies that rely on self-reported information.

There were considerable gender differences across the study populations, with a majority of participants being female. In addition, the prevalence of HV was significantly higher, in some cases twice or 2.5 times, in females than males. Therefore, the general assumption that the female gender is a significant factor in the prevalence or likelihood of having HV is confirmed by this systematic review. Roddy et al. conducted an epidemiological survey on the prevalence of HV in individuals aged above 30 years in the United Kingdom and found that it was 38% among females versus 21% among males [12].

Two studies were conducted to estimate the prevalence of HV in Japan’s elderly people and found a prevalence of 29.8% and 26.7% [13]. A similar study was conducted in Nigeria among school-going children and found a prevalence of 15.4% [14]. A study conducted on the prevalence of HV in rural Korea in 2009 found a prevalence of 64.7% [15], the highest prevalence reported among the studies included in this systematic review and meta-analysis. Age is a significant factor in HV prevalence, as observed HV levels were higher among the elderly compared to young adults and children [16-18].

Nonetheless, the findings of this review were constrained by some limitations in the studies included for analysis. One notable characteristic is the lack of a standard method of diagnosing HV among the participants, as well as the lack of a clear definition of HV. The lack of a standard method of examining HV was common across all the studies regardless of the mode of study (self-reported diagnoses and clinical observations). Most diagnoses involved examining the foot/feet for typical HV characteristics described in the studies.

Pooling was only considered for the age and gender metrics because they yielded the same results, i.e., high prevalence levels. Due to the lack of sufficient data, this review could not take into consideration other potential factors such as race, geo-location, childhood activities, and wearing of shoes.

Considering the above limitations, a large-scale epidemiological survey of HV is required. Random sampling is recommended for the survey to account for all factors, including race, geo-location, household income, genetics, and childhood activities. This review recommends the development of a standard tool/method to diagnose HV. Considering all the above limitations and recommendations, further studies on HV will provide the much-needed pool of evidence-based studies on HV and its effects on the general population. The high association between age and HV prevalence should also be considered by healthcare professionals to provide support and care to elderly patients with HV that impedes their gait stability and quality of life.

## Conclusions

This review and meta-analysis show that HV is highly prevalent among the elderly and is more likely to affect females than males. Prevalence, however, varied widely across the 11 studies analyzed, with most studies indicating a prevalence of above 25% in the general population. The causes of HV can either be biomechanical, pain, or metabolic. This review supports the long-held notion that HV is more prevalent in women than men and increases with age and that family history is a significant predictor. Limitations such as insufficient data and the lack of a standard HV diagnosis method have been considered to yield recommendations for further study.

## Appendices

**Table 2:** Search syntax used for electronic databases

PubMed
Hallux valgus/ OR Metatarsophalangeal Joint/ OR Foot deformities/ OR Foot Diseases/
Bunion$.ab,ti. OR (hallux adj5 valgus).ab,ti. OR (hallux adj5 abduct$).ab,ti. OR ((great adj5 toe) AND (deform$ or valgus or deviat$)).ab,ti. OR (foot AND deformit$).ab,ti. OR (foot problem$).ab,ti. OR chiropod$.ab,ti.
AND
Cross-Sectional Studies/ OR Case-Control Studies/ OR Prospective Studies/ OR Questionnaire$/ OR Health surveys/ OR Prevalence/ OR Risk Factors/ OR Odds Ratio/
(questionnair$ or survey or prevalence or incidence or risk$ or factor or associat$ or relationship$ or correlat$ or etiolog$ or aetiolog$ or caus$ or develop$ or predispos$ or demograph$).ab,ti.
MEDLINE
Search publications from All Years
(Map terms not exploded) “Hallux valgus”
(Keyword search: ab,ti) bunion* OR “hallux *5 valgus” OR (“great *5 toe” AND (deform* or valgus or deviat*))
AND
(Map terms not exploded) Questionnaire OR “Health survey” OR “Population Research” OR Epidemiology OR Prevalence OR Demography OR Etiology OR “Risk factor” OR Odds-Ratio OR “Disease Association”
(Keyword search: ab,ti) questionnair* or survey or prevalence or incidence or risk* or factor or associat* or relationship* or correlat* or etiolog* or aetiolog* or caus* or develop* or predispos* or demograph*
CINAHL
MH Hallux valgus OR MH Metatarsophalangeal joint OR MH Foot deformities OR MH Foot diseases
bunion* OR (hallux N5 valgus) OR (hallux N5 abduct*) OR ((great N5 toe) AND (deform* or valgus or deviat*)) OR (foot AND deformit*) OR “foot problem*”
AND
MH Cross Sectional Studies OR MH Case Control Studies OR MH Prospective Studies OR MH Questionnaires OR MH Surveys OR MH Prevalence OR MH Risk Factors OR MH Odds Ratio OR MH Descriptive Statistics
questionnair* or survey or prevalence or incidence or risk* or factor or associat* or relationship* or correlat* or etiolog* or aetiolog* or caus* or develop* or predispos* or demograph*
